# Limited HIV-2 reservoirs in central-memory CD4 T-cells associated to CXCR6 co-receptor expression in attenuated HIV-2 infection

**DOI:** 10.1371/journal.ppat.1007758

**Published:** 2019-05-16

**Authors:** Assia Samri, Charlotte Charpentier, Mariama Sadjo Diallo, Mélanie Bertine, Sophie Even, Véronique Morin, Anne Oudin, Christophe Parizot, Gilles Collin, Anne Hosmalin, Rémi Cheynier, Rodolphe Thiébaut, Sophie Matheron, Fideline Collin, Rima Zoorob, Françoise Brun-Vézinet, Brigitte Autran, the ANRS CO5 IMMUNOVIR-2 Study Group

**Affiliations:** 1 Sorbonne Université, Inserm 1135, Centre d’immunologie et des maladies infectieuses, Cimi-Paris, Paris, France; 2 IAME, UMR 1137, Inserm, Université Paris Diderot, Sorbonne Paris Cité, Laboratoire de Virologie, Hôpital Bichat, Assistance Publique-Hôpitaux de Paris, Paris, France; 3 Sorbonne-Université, Inserm 1135, CNRS ERL8255, Centre d’immunologie et des maladies infectieuses, Cimi-Paris, Paris, France; 4 Assistance Publique-Hôpitaux de Paris, Groupement Hospitalier Pitié-Salpêtrière, Département d'Immunologie, Paris, France; 5 Institut Cochin, Inserm, U1016, CNRS, UMR8104, Université Paris Descartes, Sorbonne Paris Cité, Paris, France; 6 Inserm U1219 Bordeaux Population Health, INRIA SISTM, Univ. Bordeaux, Bordeaux, France; 7 Inserm, IAME, UMR 1137, Univ. Paris Diderot, Sorbonne Paris Cité, Assistance Publique -Hôpitaux de Paris, Service des Maladies Infectieuses et Tropicales, Hôpital Bichat, HUPNVS, Paris, France; 8 University Paris7, Denis Diderot, Paris, France; 9 Sorbonne Université, Inserm 1135, Centre d’immunologie et des maladies infectieuses, Cimi-Paris, AP-HP, Hôpital universitaire Pitié-Salpêtrière, Paris, France; Leidos Biomedical Research Inc, UNITED STATES

## Abstract

The low pathogenicity and replicative potential of HIV-2 are still poorly understood. We investigated whether HIV-2 reservoirs might follow the peculiar distribution reported in models of attenuated HIV-1/SIV infections, i.e. limited infection of central-memory CD4 T lymphocytes (TCM). Antiretroviral-naive HIV-2 infected individuals from the ANRS-CO5 (12 non-progressors, 2 progressors) were prospectively included. Peripheral blood mononuclear cells (PBMCs) were sorted into monocytes and resting CD4 T-cell subsets (naive [TN], central- [TCM], transitional- [TTM] and effector-memory [TEM]). Reactivation of HIV-2 was tested in 30-day cultures of CD8-depleted PBMCs. HIV-2 DNA was quantified by real-time PCR. Cell surface markers, co-receptors and restriction factors were analyzed by flow-cytometry and multiplex transcriptomic study.

HIV-2 DNA was undetectable in monocytes from all individuals and was quantifiable in TTM from 4 individuals (median: 2.25 log_10_ copies/10^6^ cells [IQR: 1.99–2.94]) but in TCM from only 1 individual (1.75 log_10_ copies/10^6^ cells). HIV-2 DNA levels in PBMCs (median: 1.94 log_10_ copies/10^6^ PBMC [IQR = 1.53–2.13]) positively correlated with those in TTM (r = 0.66, p = 0.01) but not TCM. HIV-2 reactivation was observed in the cells from only 3 individuals. The CCR5 co-receptor was distributed similarly in cell populations from individuals and donors. TCM had a lower expression of CXCR6 transcripts (p = 0.002) than TTM confirmed by FACS analysis, and a higher expression of TRIM5 transcripts (p = 0.004). Thus the low HIV-2 reservoirs differ from HIV-1 reservoirs by the lack of monocytic infection and a limited infection of TCM associated to a lower expression of a potential alternative HIV-2 co-receptor, CXCR6 and a higher expression of a restriction factor, TRIM5. These findings shed new light on the low pathogenicity of HIV-2 infection suggesting mechanisms close to those reported in other models of attenuated HIV/SIV infection models.

## Author summary

HIV-2 induces a still poorly understood attenuated infection compared to HIV-1. We investigated whether this infection might follow peculiarities associated with other models of attenuated HIV-1/SIV infection, i.e. a limited infection of a key subset of memory CD4 T lymphocytes, the central-memory ones (TCM). Thus we studied the infection rates in peripheral blood cells from 14 untreated HIV-2 infected individuals from the ANRS-CO5 HIV-2 cohort, and found; 1) a lack of infection of monocytes, 2) extremely low infection in central-memory CD4+ T lymphocytes while HIV-2 predominated in the transitional-memory cells, 3) a poor replicative capacity of HIV-2 in individuals cells. We then investigated the cellular expression of a hundred-host genes potentially involved in HIV-2 control. We found in individuals’ TCM cells, compared to TTM ones, a lower expression of CXCR6, a potentially alternative co-receptor of HIV-2 but not of HIV-1, and a higher expression of TRIM5α, a restriction factor to which HIV-2 is more sensitive than HIV-1. Altogether our findings shed new light on the low pathogenicity of HIV-2 suggesting mechanisms close to those reported in other models of attenuated HIV/SIV infection models.

## Introduction

Human Immunodeficiency type 2 virus (HIV-2) is a Lentivirus responsible for a less pathogenic infection than HIV type 1 virus (HIV-1), characterized by slow clinical progression, prolonged maintenance of CD4 lymphocytes counts, and a high proportion of untreated individuals with undetectable plasma viral load (pVL) [1–3].

HIV-2 infection has indeed peculiar epidemiological, clinical, virological and antiretroviral susceptibility characteristics that distinguish it from HIV-1 infection [1–9]. The much slower CD4 T-cell decline [10] is in line with a preserved thymic function [11] but contrasts with the *in vitro* cytopathogenicity [12] and a relationship between CD4 T-cell depletion and immune activation that appears to be similar to that observed during HIV-1 infection [13, 14]. A main characteristic of HIV-2 infection, concentrated in Western Africa where it is presumed to infect up to 1–2 million people [15], is the low-level of circulating virus at all stages of the disease, responsible for the reduced transmissibility [16]. However, the pathophysiological mechanisms explaining these lower viral loads compared to HIV-1 remain little explored. Though close to HIV-1, HIV-2 shares only nearly 30–40% and 60% homology with HIV-1 in the Env and the Gag and Pol sequences, respectively [17], while almost identical to SIV of sooty mangabeys (sm) [18]. Robust polyfunctional anti-HIV-2 T cell responses have been associated with lower levels of viral replication, suggesting an active immune control of HIV-2 [19–23] with strong NK cells cytotoxic activity [24], comparable to what is observed in HIV-1 infected Elite Controllers. In addition, although HIV-2 uses the same CCR5 and CXCR4 co-receptors as HIV-1 [25–27], it seems to use *in vitro* a broader spectrum of alternative co-receptors (CCR1 to CCR8, CXCR6 (BONZO), GPR15 (BOB), GPR1, APJ, CX3CR1 (V28), CXCR5 and RDC1) [26–32]. Among those CXCR6 might be a more plausible HIV-2 co-receptor as it is preferentially used in aviraemic HIV-2 infected patients [33] as well as by non-pathogenic lentiviruses [34]. It also differs from HIV-1 for its susceptibility to restriction factors, as SAMHD1 [35] is not active on HIV-2, while TRIM5α, the well-known SIV restriction factor was also recently shown to limit HIV-2 but not HIV-1 production in human cells [36–38]. In addition, whether HIV-2 is sensitive to other factors such as Blimp-1, an HIV-1 restriction factor newly discovered by our group and others [39], remains unknown.

Therefore, although HIV-1 and HIV-2 infections appear to follow the same basic pathogenic mechanisms, during natural HIV-2 infection, the lower viral production, lower CD4 T-cell decline and slower progression to disease indicate a more favorable host-pathogen balance than during in HIV-1 infection. HIV-2 seems to be able to spread and build a reservoir of infected cells like HIV-1. HIV-2 DNA levels in peripheral blood mononuclear cells (PBMCs) are equivalent to those measured in HIV-1 infected individuals with similar CD4 cell counts [40–43]. The divergence between the levels of viral DNA in PBMCs and of plasma RNA between HIV-2 and HIV-1 infection suggests a scenario with a first phase of active virus replication during primary infection, during which the viral reservoirs are established, followed by a second phase of viral replication control. Indeed, active infection in the early stages of HIV-2 infection is also suggested by the high immune activation and peripheral plasmacytoid dendritic cell (pDC) depletion found in different studies [13, 44]. However, the control of HIV-2 replication might occur soon enough before immune functions are definitively damaged.

During HIV-1 infection, the latent reservoir of integrated but inducible HIV-1 predominantly takes place in resting central-memory T (TCM) lymphocytes CD4 [45, 46] and in few monocytes/macrophages [47, 48]. Conversely, in the classical sooty-mangabey model of attenuated SIV infection, the TCM reservoirs are limited compared to more differentiated cells and this is related to low CCR5 co-receptor expression [49]. Supporting the low TCM infection level as a hallmark of reduced pathogenicity, the TCM from HIV-1 infected Long Term Non Progressors (LTNP) with the protective HLA-B*27 or B*57 alleles are also less infected than transitional-memory CD4-T-cells (TTM) [50]. In addition, a similar low TCM cell contribution to the HIV-1 reservoirs is characteristic of HIV-1 infected post-treatment controllers [48]. Nevertheless, whether the same peculiar reservoir distribution in resting CD4+ memory T cells is true in the low pathogenic HIV-2 infection remains unknown.

To define better the characteristics of the HIV-2 reservoirs, we analyzed the latent and inducible blood reservoir of HIV-2 and its distribution among CD4+ peripheral blood cells from individuals followed in the French ANRS HIV-2 CO5 cohort [51] whose adaptive and innate immune responses are being characterized in the context of the ongoing Immunovir reservoir study [20, 52]. We particularly investigated whether monocytes are infected *in vivo* and tested the hypothesis of a limited infection of TCM, like in non-pathogenic SIVsm infection or in the non-progressive HIV-1 infection of HLA-B*27^+^ or B*57^+^ LTNPs.

## Results

### Characteristics of studied HIV-2 infected individuals

This HIV-2 reservoir study was performed on PBMC from 14 HIV-2 infected individuals from the ANRS-CO5 HIV-2 Cohort, identified as non-progressors (n = 12) according to our previous studies [51] or progressors (n = 2), and defined in material and method section. These individuals were previously described in Angin et al. and Lucar et al. [20, 52]. As described in Table 1, 8 out of 14 (57%) were women and except for individual 11, all donors were from Western Africa. At inclusion, the median age was 51 [IQR: 44–55] years; the median duration of known infection was 12.6 [IQR: 9.1–22.8] years and median CD4 T-cell count was 966 cells/μL [IQR: 820–1216]. Four individuals displayed an HLA-B*27, B*57 or B*58 allele. Five individuals had detectable pVL under the limit of quantification (LOQ) at 1.3, 2.4, 5.6, 19, and 58 copies/mL using an ultrasensitive assay. HIV-2 viral group was A in 5 individuals, B in 7 individuals, and not available because of amplification failure for the two remaining individuals. Median total HIV-2 DNA in PBMCs was above the LOQ in 13 individuals with a median of 1.94 log_10_ copies/10^6^ PBMC [IQR: 1.53–2.13] for a median total DNA amount analyzed per PCR well of 463 ng [IQR: 328–608]. HIV-2 DNA levels did not differ between the four HLA-B*27, B*57 or B*58 individuals 2.02 log_10_ copies/10^6^ PBMC [IQR: 1.15–2.12] and the others 1.73 log_10_ copies/10^6^ PBMC [IQR: 1.34–2.16] (p = 0.94).

**Table 1:** Participants characteristics.

Participants	Gender	Age (years)	CD4 T-cell Counts (cells/mm^3^)	Time since diagnosis (years)	Ultra Sensitive Viral Load (copies/mL plasma)	HIV-2 DNA (copies/10^6^ PBMC)	HIV-2 DNA (log_10_ copies/10^6^ PBMC)	HIV-2 group	HLA-A	HLA-B	HLA-C	Geographic Origin
1	M	52	891	11.3	<1	131	2.12	B	02/03	49/57	07/18	Ivory Coast
2	M	53	1228	17.6	<1	170	2.23	A1	23/23	07/14	07/08	Guinea Bissau
3	F	52	604	25.6	19	139	2.14	B	02/23	15/52	02/16	Guinea Conakry
4	M	57	1090	23	ND	107	2.03	A1	23/34	53/53	04/04	Ivory Coast
5	F	34	1212	12.5	5.6	88	1.94	A1	03/26	58/58	03/07	Gambia
6	F	50	1776	8.8	<1	53	1.73	B	68/68	07/52	-	Ghana
7	F	39	1036	12.7	<1	53	1.73	B	03/03	35/53	04/04	Ivory Coast
8	F	48	859	9.2	ND	40	1.60	B	34/34	15/53	02/04	Guinea Conakry
9	F	49	895	11.3	<1	28	1.45	B	02/68	15/51	14/16	Ivory Coast
10	F	54	1118	27.2	2.4	10	0.99	B	03/74	14/15	07/08	Guinea Conakry
11	M	59	1300	8.8	<1	<7.5	<0.88	NA	01/29	44/57	06/16	Colombia
12	M	44	707	20.5	1.3	187	2.27	A	03/23	35/53	04/04	Ivory Coast
13#	F	43	858	22.7	<1	10	1.00	NA	1/33	15/35	04/14	Ivory Coast
14#	M	70	502	7.4	58	127	2.10	A	02/02	27/53	02/04	Guinea Conakry
Median		51	966	12.6	1	70.5 (88*)	1.84 (1.94*)					
[IQR 25–75%]		[44–55]	[820–1216]	[9.1–22.8]	[1–4]	[34–135] *	[1.53–2.13] *					

# progressors individuals; ND: Not Done; NA: Not Amplifiable;

* in individuals with detectable HIV-2 DNA

### Distribution of CD4 cells subsets and CCR5 expression in HIV-2 infected individuals

**Fig 1:**
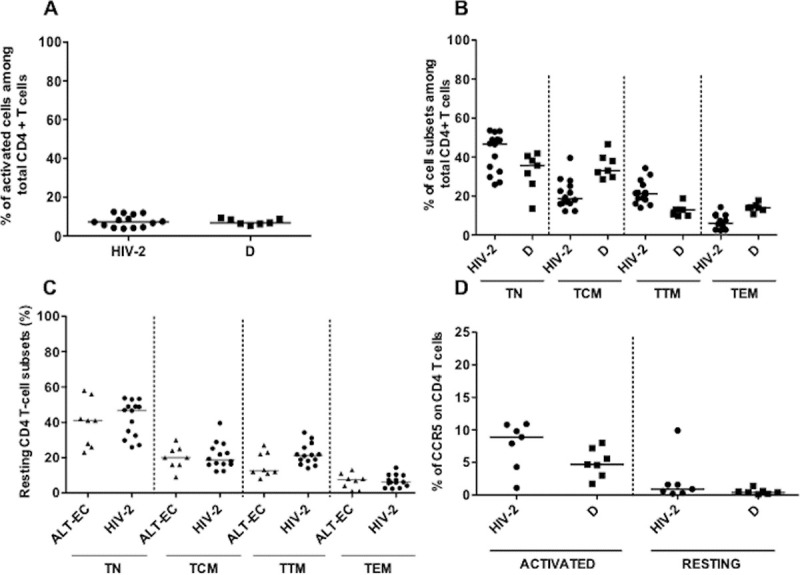
**A**) Percentage of activated CD4 T lymphocytes (left panel) of 14 HIV-2 infected subjects (dots) compared to 7 donors (D) (squares). Each symbol represents a subject and the medians are shown. **B**) The repartition of resting CD4 T-cell subsets was assessed in 14 HIV-2 infected subjects (dots) and in 7 donors (D) (squares). The analyzed resting CD4+ subsets are: naive (TN; CD45RA+CCR7+CD27+) central-memory (TCM; CD45RA-CCR7+CD27+) transitional memory (TTM; CD45RA-CCR7-CD27+) and effector-memory cells (TEM; CD45RA-CCR7-CD27-). Each symbol represents a subject and the medians are shown. **C**) Resting CD4 T-cell subsets of 7 HIV-2 infected subjects (dots) compared to 8 HIV-1 infected Long-Term-Non-Progressors (ALT) (triangles). The analyzed resting CD4+ subsets are TN, TCM, TTM and TEM. Each symbol represents a subject and the medians are shown. **D**) Expression of CCR5 on CD4 T-cells activated and resting CD4 T-cells from 7 HIV-2 infected subjects (dots) compared to 7 donors (D) (squares). Each symbol represents a subject and the medians are shown.

The proportions of activated CD4 T-cells (Fig 1A) or monocytes were not significantly different in HIV-2 infected individuals compared to HIV-negative donors (D). Similarly, there were no significant differences between the proportions of TN (CD45RA+CCR7+CD27+ cells: 46.76% vs 35.72%) compared to HIV-negative D, However, differences were observed for TCM (CD45RA-CCR7+CD27+ cells: 18.63 vs 33.01%; p = 0.001), TTM (CD45RA-CCR7-CD27+ cells: 21.25% vs 12.87%; p = p = 0.001), TEM (CD45RA-CCR7-CD27- cells: 6.08% vs 13.94%; p = 0.001) (Fig 1B) subsets among CD4 T-cells compared to HIV-negative D. In addition, no differences were observed when comparing the frequencies of subsets among resting CD4 T-cells from the HIV-2 infected individuals to those of 8 HIV-1 infected ALT Elite Controllers (ALT EC) (Fig 1C). The surface expression of CCR5 did not differ between HIV-2 infected individuals and in D in activated or resting CD4 T-cells (Fig 1D) nor on the various resting CD4 T-cell subsets (S1 Fig).

### Preferential distribution of HIV-2 reservoirs in CD4+ TTM and lack of monocyte HIV-2 infection

The distribution of total HIV-2-DNA was quantified in various populations sorted from the PBMC of the 14 individuals. Cells were sorted into CD3-CD4-CD14+ Monocytes, CD25+CD69+HLADR+ activated CD4 T-cells and CD25-CD69-HLADR- resting CD4 T-cells sub-sorted into TN, TCM, TTM, and TEM. After sorting, HIV-2 DNA was quantified in median total DNA 312 ng [IQR: 171–559]. Regarding the TEM cell subset, as could be expected; the number of sorted TEM cells tested for HIV-2 DNA quantification was lower than other subsets in most patients and DNA levels were below 200 ng/PCR in 12 out of 14 individual samples (S1 Table).

**Fig 2:**
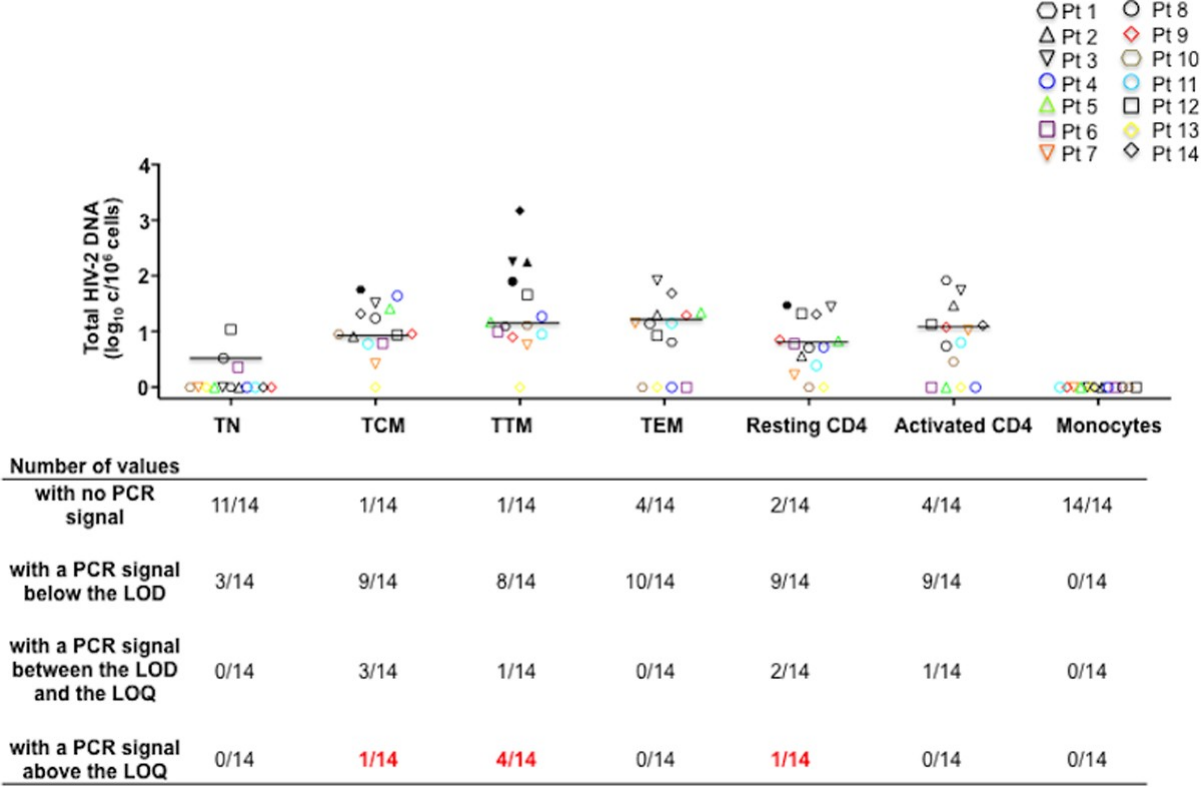
The distribution of total HIV-2 DNA was quantified in various sorted populations from peripheral blood mononuclear cells (PBMCs) from 14 HIV-2 infected subjects. Total HIV-2 DNA quantification in CD3-CD4-CD14+ Monocytes, CD25+CD69+HLADR+ activated CD4 T-cells and CD25-CD69-HLADR- resting CD4 T-cells sub-sorted into: naive (TN; CD45RA+CCR7+CD27+) central-memory (TCM; CD45RA-CCR7+CD27+) transitional memory (TTM; CD45RA-CCR7-CD27+) and effector-memory cells (TEM; CD45RA-CCR7-CD27-). Results are expressed as the log_10_ HIV-2 DNA copies per million cells, and medians are shown. Each symbol represents a subject. Filled symbols show quantifiable DNA detection. The limit of detection (LOD) of the HIV-2 DNA assay is 3 copies/PCR and the limit of quantification (LOQ) is 6 copies/PCR.

**Fig 3:**
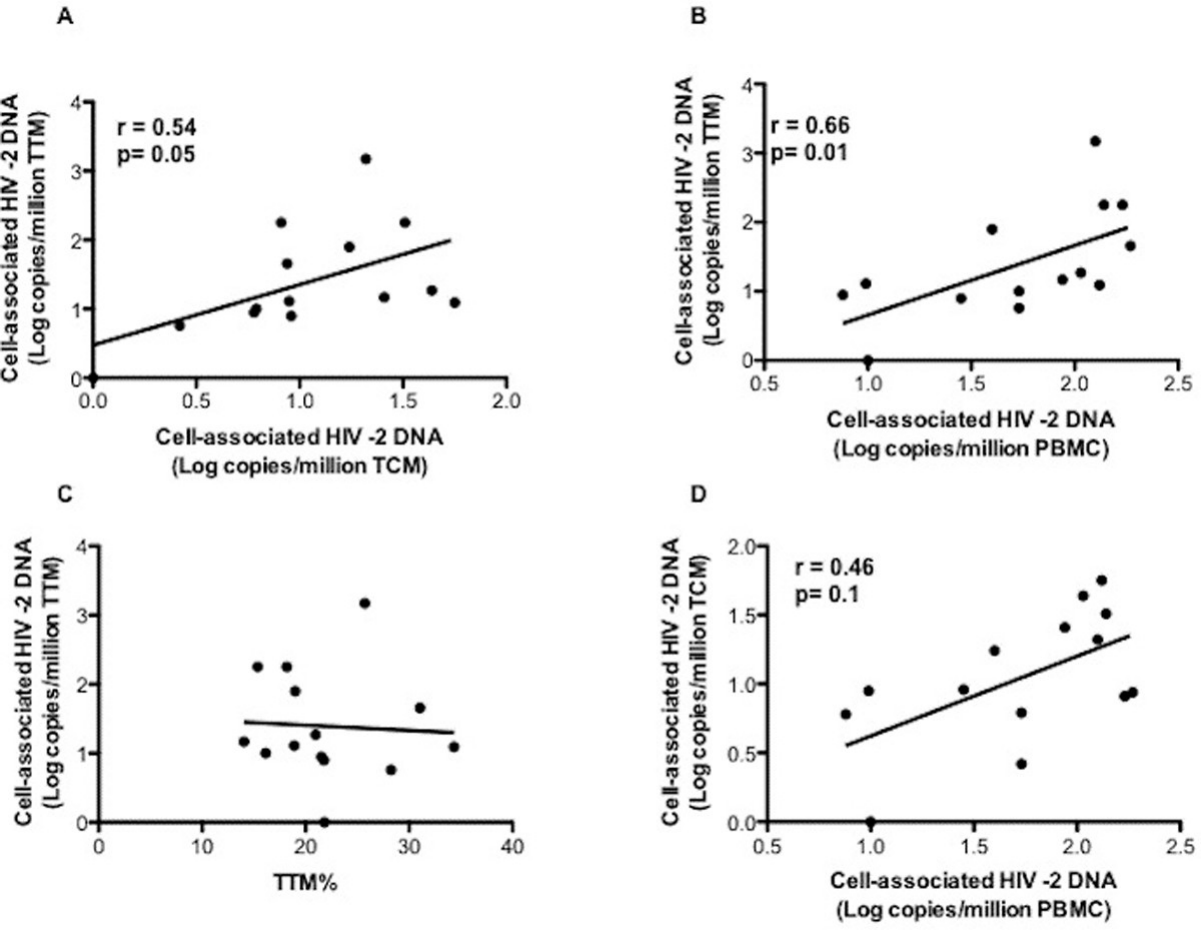
Relationship between cell-associated HIV-2 DNA in transitional memory T cells (TTM; CD45RA-CCR7-CD27+) and **A)** cell-associated HIV-2 DNA in central-memory (TCM; CD45RA-CCR7+CD27+) and **B)** cell-associated HIV-2 DNA in peripheral blood mononuclear cells (PBMCs) and **C)** transitional memory T cells (TTM; CD45RA-CCR7-CD27+) numbers in the peripheral blood. **D)** Relationship between cell-associated HIV-2 DNA in central-memory (TCM; CD45RA-CCR7+CD27+) and in in peripheral blood mononuclear cells (PBMCs). Fourteen HIV-2 infected subjects are represented. Each symbol represents a subject. Correlations were determined with Spearman's rank test.

**Fig 4:**
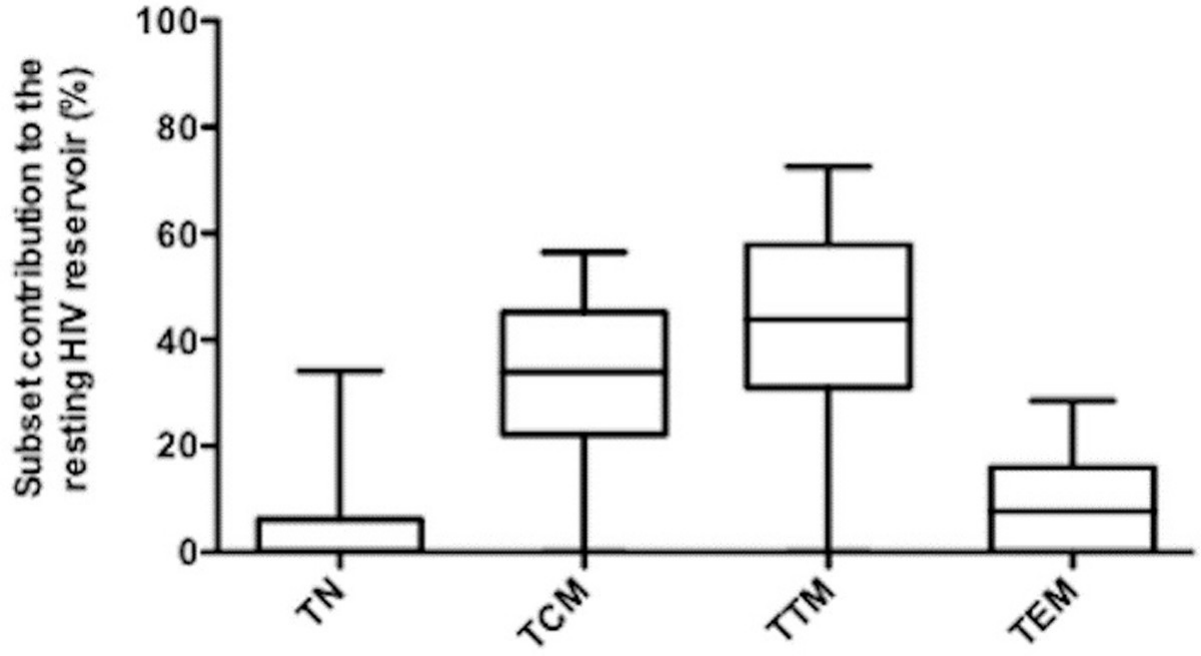
The CD4 T-cell subsets contribution to the resting HIV-2 reservoir considering both infection levels and frequency. Resting CD4 T-cells: naive (TN; CD45RA+CCR7+CD27+) central-memory (TCM; CD45RA-CCR7+CD27+) transitional memory (TTM; CD45RA-CCR7-CD27+) and effector-memory cells (TEM; CD45RA-CCR7-CD27-) are represented from 14 HIV-2 infected subjects. The results are expressed as the median percentage of the resting CD4 T-cells HIV-2 reservoir, with interquartile range [25%–75%] and minimum and maximum values.

HIV-2 DNA was consistently undetectable among the median 1.36 million [IQR: 0.88–1.71] monocytes sorted from each individual. In contrast HIV-2 DNA was detectable in activated and resting CD4 T-cells from 10 and 11 individuals, respectively (Fig 2) but were quantifiable only in the resting CD4 T-cells from a single individual (Pt) (Pt 1: 1.47 log_10_ copies/10^6^ cells). Finally, HIV-2 DNA could be quantified only in the TTM from four individuals (Pt 2; Pt 3; Pt 8; Pt 14 with 2.25, 2.25, 1.90, and 3.17 log_10_ copies/10^6^ cells respectively) (S1 Table), while in the TCM from only one individual (Pt 1; 1.75 log_10_ copies/10^6^ cells) (S1 Table). However, no significant difference was observed between TTM and TCM qualitatively (p = 0.37) or quantitatively (p = 0.1). Overall, these low circulating HIV-2 DNA levels were quantifiable in CD4 subsets from only 5 of the 14 individuals tested, 2 of whom only having also quantifiable plasma viremia. The two individuals with the highest ultrasensitive pVL (58 and 19 copies/mL) also had detectable TTM reservoirs, the third was untested, the fourth was undetectable. HIV-2 DNA levels in TTM cells showed a trend towards a correlation with the TCM infection levels (p = 0.05; r = 0.54) (Fig 3A) and were correlated to HIV-2 total DNA in PBMC (p = 0.01; r = 0.66) (Fig 3B) while HIV-2 DNA levels in TCM were not (Fig 3D). Of note HIV-2 DNA levels in TTM were not correlated to the TTM cell numbers in the peripheral blood (Fig 3C). This preferential HIV-2 DNA distribution among resting CD4 TTM cells was not associated with the hosts’ HLA-B*27, *57 or B*58 alleles. We then evaluated the relative contribution of each subset to the whole pool of infected resting CD4 T-cells by taking into account the infection level and the frequency in blood of each subset (Fig 4), showing that the TTM subset contributed to 46% of the total infected resting CD4 T-cell pool and the TCM only to 33% (Fig 4). However, no significant difference, in term of contribution, was observed between TTM and TCM (p = 0.108).

### Poor inductibility of HIV-2 in CD4+ peripheral blood cells

**Fig 5:**
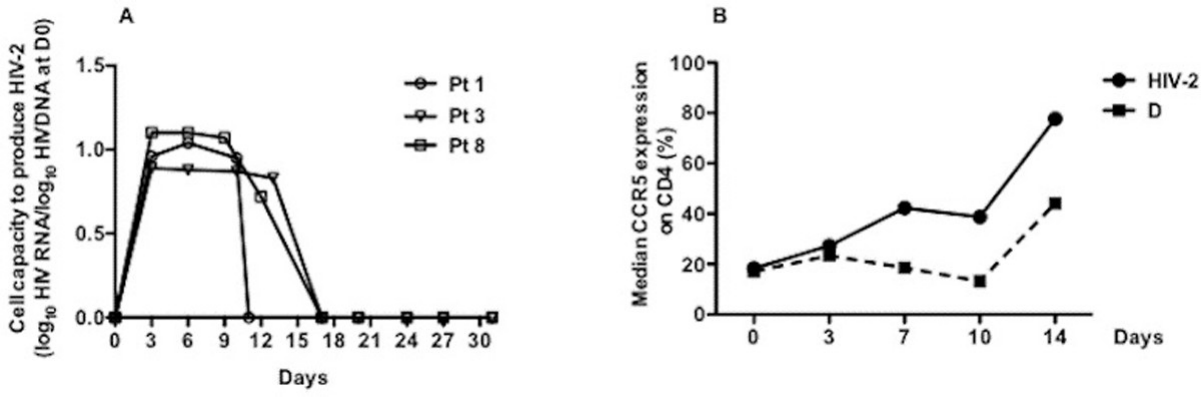
**A)** *In vitro* inducibility of HIV-2 reservoir in CD8-depleted peripheral blood mononuclear cells (PBMCs). Cell capacity to induce HIV-2 replication was evaluated in 11 HIV-2 infected subjects by stimulating CD8-depleted PBMC with anti-CD3/anti-CD28 co-stimulation plus IL-2, plus IL-7 plus IL-15. Allogeneic feeder CD8-depleted PBMC from a donor were added to culture at D13. Culture supernatants were collected every three days for HIV-2 viral load quantification from D0 to D31. Results are expressed as the log10 of the ratio between the numbers of HIV-RNA copies quantified on a given day of culture and the level of total HIV-DNA in the subset measured at D0 of culture. Each symbol represents a subject. **B)** Expression of CCR5 in culture on CD4 T-cells from 7 HIV-2 infected subjects (dot) compared to 7 donors (D) (square). Median expression of CCR5 on CD4 T-cells during 14 days of culture is represented.

The capacity to induce HIV-2 replication from CD8-depleted cells was evaluated after strong TCR stimulation by anti-CD3/anti-CD28 coated beads, IL-2, IL-7 and IL-15; and allogeneic cells at D0 of culture. The production of HIV-2 RNA was detected in 3 of the 11 tested samples between D3 and D15 in supernatants with low levels ranging between 2 and 3 log_10_ HIV-2 RNA copies/mL (Fig 5A). Interestingly, the three individuals from whom HIV-2 was inducible *in vitro* had the highest levels of HIV-2 DNA quantified in TTM (Pt 3 and Pt 8) or in TCM (Pt 1).

In order to better understand such low virus production, we analyzed the expression of the CCR5 co-receptor during culture. As expected (Fig 5B), CCR5 expression increased steadily from D3 to D14 in CD4 T-cells from HIV-2 infected individuals reaching even higher levels than in D.

### Distribution of HIV-2 DNA among memory CD4 cell subsets is associated with an imbalance in TRIM5 and CXCR6 gene expression

**Fig 6:**
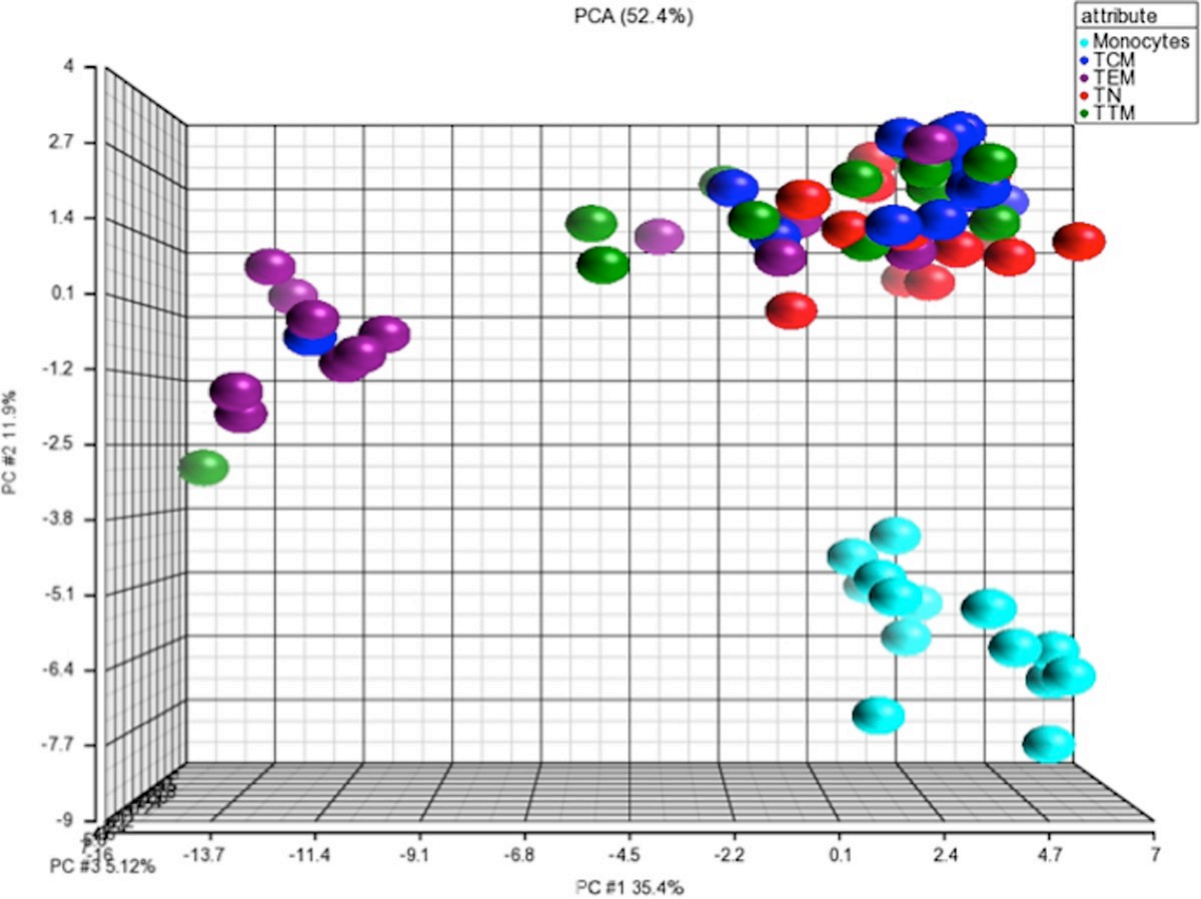
Transcriptomic analysis: A broad multiplex transcriptional profiling analysis of 96 genes was performed in monocytes (CD3-CD4-CD14+) and resting CD4 T-cells subsets: Naive (TN; CD45RA+CCR7+CD27+) central-memory (TCM; CD45RA-CCR7+CD27+) transitional memory (TTM; CD45RA-CCR7-CD27+) and effector-memory cells (TEM; CD45RA-CCR7-CD27-) from 14 HIV-2 infected subjects. A principal component analysis (PCA) plot representation of gene expression profile in each population is shown.

In order to better understand the determinants of the atypical distribution of HIV-2 reservoirs among memory CD4 T-cells we performed a broad multiplex transcriptional profiling analysis of 96 genes in each resting CD4 T-cell subset (TN; TCM; TTM; TEM) and monocytes sorted from the 14 HIV-2 infected subjects PBMC. These genes were mainly related to sensing, inflammation, cytokines, chemokines, interferon signalization pathways, with a particular focus on the putative alternative HIV-2 co-receptors like CXCR6 (S2 Table) and restriction factors such as TRIM5α and Blimp-1. We first excluded that Blimp-1 could act as an HIV-2 restriction factor by analyzing the HIV-2 LTR sequences. In contrast to HIV-1, they do not contain any Blimp-1 binding sites. Then a principal-component analysis (PCA) of the multiplex transcriptional raw data (Fig 6) allowed us to distinguish monocytes from all resting CD4 T-cell subsets. Among the latter the TEM cells displayed a different gene profile, characteristic for more advanced differentiation compared to the three other TN, TCM and TTM populations.

**Fig 7:**
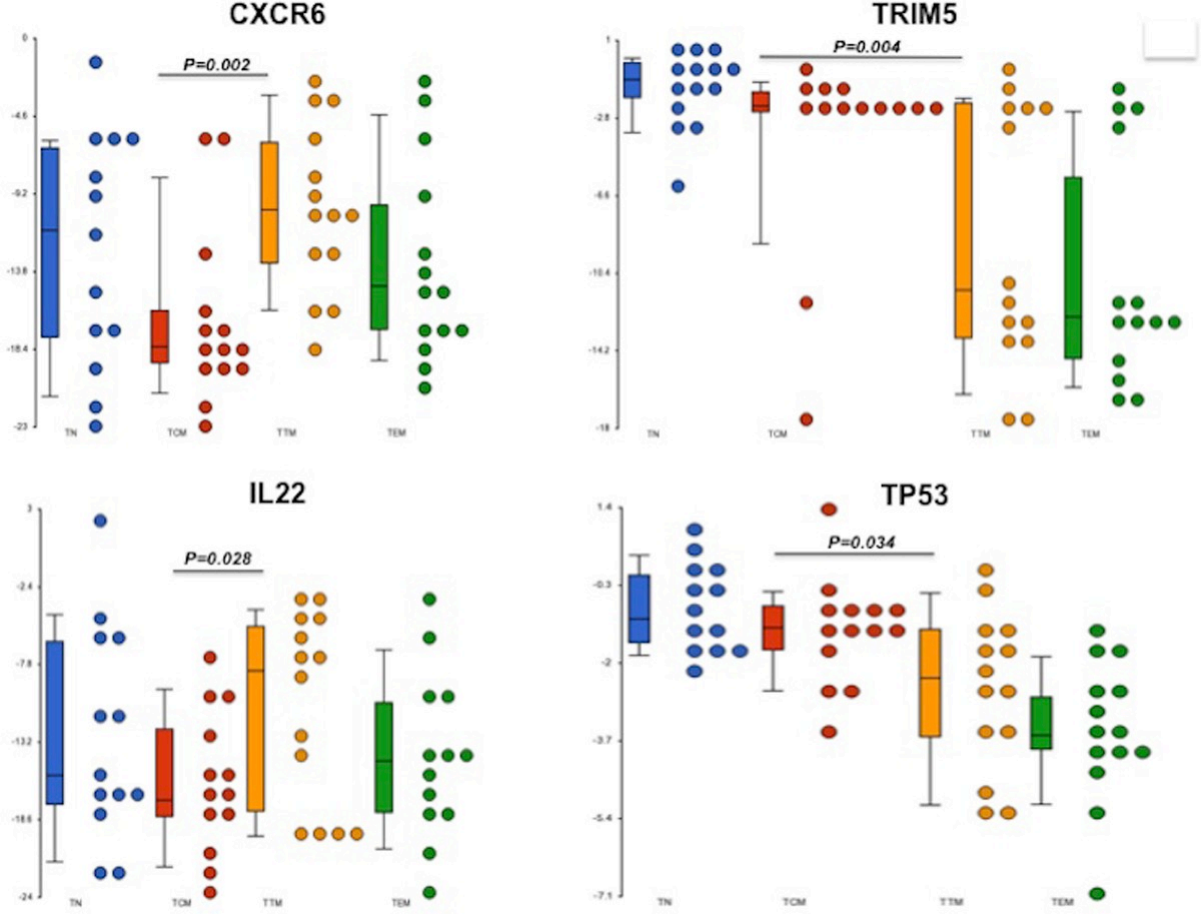
Transcriptomic analysis: A broad multiplex transcriptional profiling analysis of 96 genes was performed in monocytes (CD3-CD4-CD14+) and resting CD4 T-cells subsets: Naive (TN; CD45RA+CCR7+CD27+) central-memory (TCM; CD45RA-CCR7+CD27+) transitional memory (TTM; CD45RA-CCR7-CD27+) and effector-memory cells (TEM; CD45RA-CCR7-CD27-) from 14 HIV-2 infected subjects. The relative expression of CXCR6, TRIM5, IL22 and TP53 in resting CD4 T-cells subsets. A two-tailed Wilcoxon matched-pairs signed rank test was used to compare cell subsets frequencies, CXCR6, TRIM5, IL22 and TP53 expression within TCM and TTM.

As HIV-2 DNA was detectable only in the TTM and TCM subsets, we focused our analysis on these two subsets. Among the 96 genes studied only four genes significantly differed between those two subsets. CXCR6, a putative alternative HIV-2 co-receptor and IL-22, an inflammatory cytokine, were 94 (p = 0.002) and 37-times (p = 0.028) more expressed in TTM than in TCM. In contrast, TRIM5α, a SIV/HIV-2 potential restriction factor for HIV-2 but not HIV-1, and TP53, a tumor suppressor, were 35-times (p = 0.004) and 2-times (p = 0.034) less expressed in TTM than in TCM (Fig 7). However, the significances observed for these four genes were lost after FDR correction. The same analyses done on sorted cells from the 4 donors with the same African origin (West Africa) did not show differences compared to the infected subjects

### Imbalance in CXCR6 and TRIM5α protein expression between TCM and TTM

In order to further investigate whether the differences in CXCR6 and TRIM5 gene expression could be observed at the protein level in CD4 T-cell subsets, we performed an additional flow cytometric analysis of CXCR6 cell surface and TRIM5α intra-cellular expression in CD4 T-cell subsets from 12 HIV-2 infected individuals, and from 9 donors (D) (4 French and 5 West-African (A)).

**Fig 8:**
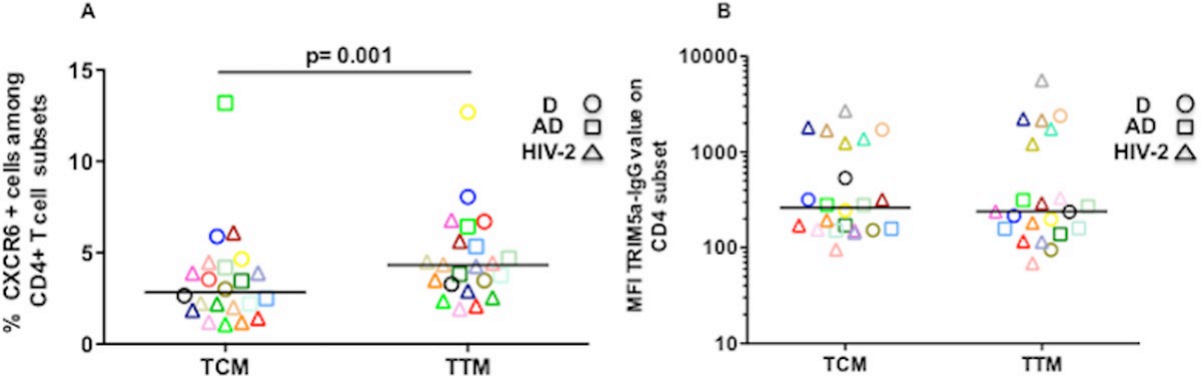
Flow-cytometry analysis of CXCR6 and TRIM5α proteic expression in TCM and TTM CD4+ T cells Flow cytometry analysis of central-memory (TCM; CD45RA-CCR7+CD27+) and in transitional memory (TTM; CD45RA-CCR7-CD27+) CD4+ T-cells subsets from 12 HIV-2 infected subjects (open triangle), 4 donors (D) (open dot) and 5 West-African donors (AD) (open squares) showing the **A)** Cell surface expression of CXCR6. **B)** Mean fluorescence intensity (MFI) of intra-cellular TRIM5α after subtracting the secondary immunoglobulin MFI. Each symbol represents a subject and the medians are shown.

We first observed that TCM display less CXCR6+ cells than TTM with a highly significant difference (median 2.8% versus 4.3%, respectively; p = 0.001) (Fig 8A). This difference is also observed in HIV-2 patients with a significant difference (median 2.1% versus 3.9%, respectively; p = 0.005). These differences followed the known CCR5 gradient between TCM and TTM with approximately one third of CXCR6+ TCM and TTM cells displaying CCR5.

As TRIM5α expression among CD4 T-cells had never been examined to our knowledge by flow cytometry either in normal or in HIV-infected individuals, we first developed an intra-cellular indirect staining of TRIM5α that showed a weak but highly reproducible expression on all CD4 T-cells and all subsets (S2 Fig). The mean fluorescence intensities were similar in CD4 T-cells from HIV-2 infected and D. We then focused on the comparison between TCM and TTM but found no significant differences in TRIM5α expression between TTM and TCM cells, even after subtracting the secondary immunoglobulin MFI (median MFI = 239 and 263, respectively) (Fig 8B).

## Discussion

Our study provides the first immunological characterization of the HIV-2 peripheral blood cell reservoirs in individuals infected by HIV-2 with a poorly productive HIV-2 infection and a slow or absent disease progression. This analysis, despite of limited samples size, clearly demonstrates that HIV-2 reservoirs have different characteristics from the HIV-1 reservoirs by a total lack of monocytic infection and a strong trend towards a predominance of the infection in TTM instead of TCM cells. This later point might reflect the differential expression of CXCR6 an alternative HIV-2 co-receptor, and of the restriction factor Trim5α among those two CD4 memory cells subsets.

Overall, these HIV-2 infected individuals had low circulating HIV-2 DNA levels, quantifiable in only 5 of the 14 individuals tested. HIV-2 was reactivable *in vitro* in PBMCs from only 3 individuals samples but this weak *in vitro* growth was not related to the HIV-2 DNA burden. This low HIV-2 *in vitro* production did not reflect a bias in the assay we used. Indeed since the HIV-2 DNA amounts were low and the replication cycle of HIV-2 is longer than that of HIV-1, the assay was adapted by culturing for 30 days culture instead of the 6–14 days as for HIV-1 [47, 48, 50] and by adding allogeneic feeder cells to the strong T cell stimuli used for HIV-1 cultures. Our results confirm the known low productivity of HIV-2 but might reflect the individuals’ characteristics, as 12 out of the 14 individuals were non-progressors and the two individuals with progressive disease requiring initiation of antiretroviral therapy had the same low level of HIV-2 reservoirs and limited *in vitro* reactivation for one tested individual. This suggests that pathogenicity might be independent from the HIV-2 growth in human PBMCs.

Two key points emerged when analyzing the distribution of HIV-2 DNA among the various mononuclear subsets. First, HIV-2 DNA was never detectable among individuals’ monocytes, while several studies show that these cells are infected, although at low frequencies, by HIV-1 [47, 48, 50]. We cannot entirely exclude that such inability to detect HIV-2 DNA in monocytes might reflect the low-levels of HIV-2 reservoirs but this key point did not reflect a paucity of monocytes tested since we analyzed about 1 million monocytic cells per individual, an amount that was far above the sorted CD4 T-cell numbers in which we detected HIV-2. This means that if monocytes were infected by HIV-2, it would be with a frequency lower than one in a few million cells. The lack of HIV-2 infection we detected *ex vivo* in individuals monocytes point had never been reported so far. It is surprising when considering that HIV-2 expresses vpx, a regulatory protein which counteracts the restriction factor SamHD1 at least when introduced within a pseudotyped virus [53]. However, it is in accordance with the lack of *in vitro* HIV-2 infection in cultured monocytes-derived dendritic cells [54]. Although we had no access to tissue samples and cannot extrapolate to macrophage infection, our results demonstrate that monocytes do not constitute a reservoir for HIV-2, which is purely concentrated among CD4 T lymphocytes at least in the peripheral blood. Furthermore, one of the intrinsic limitations of this study could be the limited amount of cells in some subsets, especially in TEMs that were rare in those non-progressors. However, in most cases the amount of DNA was sufficient to validate the assay.

The second major point is that the distribution of HIV-2 reservoirs among memory CD4 T cell subsets differ from the usual reservoir distribution observed in HIV-1 infection and suggest our working hypothesis of a limited reservoir in TCM is in line with what reported in the sootey-mangabey model of attenuated though productive SIV_sm_ infection [49], as well as, with our own group’s description of a relative TCM subset protection in HIV-1 infected LTNPs bearing the protective HLA-B*27 or B*57 alleles [50] or in post-treatment HIV controllers [48]. Altogether these results suggest the relative protection of TCM cells might be an attribute of low pathogenicity models of the HIV/SIV infections.

Our mechanistic exploration first showed such atypical reservoir distribution was totally unrelated to the HLA alleles associated with HIV-1 non-progression, in line with the fact that no clear HLA alleles had been related to HIV-2 non-progression. These characteristics also did not reflect a particular distribution of CCR5, as reported in the sm model [49]. Indeed CCR5 expression followed here-in the same progressive gradient of expression among memory CD4 T-cells, with a slightly lower expression on TCM than on TTM, as observed during HIV-1 infection while the CCR5 expression normally increased on activated CD4 T-cells from HIV-2 infected individuals. We also excluded a possible role of Blimp-1 in TCM as reported in Elite Controllers since HIV-2 has no binding sites to this transcriptional repressor acting on HIV-1 [39, 55]. Instead the multiplex transcriptional exploration of a hundred genes including co-receptors, restriction factors or various ISGs clearly demonstrated that two molecular mechanisms might play a role in this preferential distribution of the HIV-2 cellular reservoir. First, a significantly lower expression of the CXCR6 gene in TCM than in TTM, which was confirmed at the protein level, might contribute to the peculiar HIV-2 distribution, as CXCR6 has been proposed as an alternative co-receptor for HIV-2 but not HIV-1 [33]. Indeed, several studies reported the use *in vitro* of a broader spectrum of alternative co-receptors by HIV-2 [33, 56, 57] with contradictory findings regarding the association between the use of these several possible alternative co-receptors and the HIV-2 pathogenicity. Furthermore, the CXCR6 usage has been recently shown to characterize non-pathogenic lentiviruses [34] and that the CXCR6 usage is abrogated by a proline at position 326 of the gp105 V3 loop. Interestingly, among the 259 HIV-2 gp105 sequences available from the ANRS-HIV-2 cohort, none of them displayed a proline at this position (C Charpentier and F Brun-Vezinet, personal communication). Thus, our data reinforce the findings that the CXCR6 expression may contribute, among other things, to the attenuation of HIV-2 infection. Second, a significantly higher expression of the TRIM5α gene in TCM compared to TTM might also contribute to this distribution, although the transcripts differences in the HIV-2 infected individuals population and do not reach significance in controls and are not detectable at the proteic level. Indeed TRIM5α acts as a potent restriction factor for SIV but not for HIV-1. Thanks to the close similarity between SIVsm and HIV-2, all HIV-2 capsid sequences express high levels of susceptibility to hTRIM5α [58], a property that might contribute in part to the lower replication in TCM compared to TTM. Altogether the CXCR6 characteristics and, though at a lesser level, the Trim5α ones, might contribute to modify the susceptibility to infection of the various memory CD4 T-cell compartments to HIV-2 compared to HIV-1.

In conclusion, two series of attributes characterize the distribution of the HIV-2 reservoirs among peripheral blood mononuclear cells of non or slow-progressing HIV-2 infected individuals. First, the lack of HIV-2 infection in monocytes suggests that this SIV-derived virus is poorly adapted to the myeloid compartment. Second, the low HIV-2 infection of central-memory CD4 T-cells appears to reflect the cellular distribution of the CXCR6 co-receptor and of restriction factors proposed to act on HIV-2 but not on HIV-1, suggesting HIV-2 might also be less adapted to human central-memory CD4 T cells than to transitional-memory ones. Altogether our findings suggest this peculiar distribution of the HIV-2 reservoirs might be related to key host factors, thus shedding new light on the still poorly understood low pathogenicity of the HIV-2 infection.

## Materials and methods

### Ethics statement

This study was conducted according to the principles expressed in the Declaration of Helsinki. The study was approved by the ethics committee (i.e., Comité de Protection des Personnes of Ile de France XI). All study participants were adults and provided written informed consent for the collection of samples and subsequent analyses.

### Participants

Study was conducted in 14 HIV-2 infected individuals, 12 HIV negative donors and 8 HIV-1 infected LTNPs.

They were 14 participants of the ANRS IMMUNOVIR-2/RESERVOIR study (part of the French ANRS HIV-2 CO5 cohort) which included asymptomatic treatment-naive individuals infected with HIV-2 infection alone, divided in two groups non progressors (n = 12) (HIV-2 diagnosis since at least 8 years, with at least three CD4 cell count or pVL measures during the 5 last years, CD4 cell count >500/μl since at least 5 years without a rapid decrease CD4 cell count slope (i.e. >50 cells/year) during the 3 last years) [51]: and progressors (n = 2) (with CD4 cell count <350/μl or rapid CD4 cell count slope decrease (i.e. >50 cells/year) during the 3 last years or CD4 cell count <500/μl for at least 36 months or CD4 cell count <500/μl and HIV-2 pVL>100 copies/mL) (Table 1); and West-African donors (AD) (n = 5) (i.e. HIV-negative) served as controls were matched for geographic origin, sex and age.

As controls 8 HIV-1 infected LTNPs from the ANRS-CO15 ALT cohort [50, 59] were included with the following characteristics: 3/8 women, median age 40 [IQR: 37–44] years, infected since a median 11 [IQR: 10–13] years, with median CD4 cell counts of 809/μL [IQR: 707–905], pVL of 69 copies/ml [IQR: 49–96] and cell-associated total HIV-1 DNA of 19 copies [IQR: 13–63] /10^6^ PBMC. In addition 7 donors (D) from the French Blood Bank (Etablissement Français du Sang) were studied.

For all participants, PBMC were isolated by Ficoll density gradient and cryopreserved in liquid nitrogen until use.

### Ultrasensitive HIV-2 plasma viral load (pVL) quantification

An ultrasensitive pVL method was used. The maximum volume of available plasma was centrifuged (23 600 g, 2H). After elimination of the supernatant, the pellet was resuspended in 1mL of RPMI (Thermo Fisher Scientific, Waltham, MA, USA), then extracted with the MagNa Pure LC Total Nucleic Acid (Roche, Mannheim, Germany). The pVL was determined using the Generic HIV-2 viral load assay (Biocentric, Bandol, France) showing good sensitivity and accuracy to quantify HIV-2 A and B groups as previously described [60]. Thus, for example, if the initial total amount of plasma was 10 mL, the LOQ became 4 copies/mL.

### HIV-2 viral group and tropism determinations

HIV-2 viral group and tropism were genotypically determined as previously described [61, 62].

### Sorting of peripheral blood CD4+ cell subsets

Eighty millions cryopreserved PBMC were thawed with a viability above 80% and were depleted from CD8 cells using magnetic beads (Miltenyi, Biotec), and a mean of 42 millions CD8- cells per subject were sorted after staining with the following antibodies: anti-CD3-Pacific Blue, anti-CD4-AlexaFluor700, anti-CCR7-PE-Cyanine7, anti-CD27-APC, anti-CD69-FITC, anti-HLA-DR-FITC, anti-CD14-BV711 (BD-Bioscience, San-Jose, California, USA), anti-CD45RA-ECD, anti-CD25-FITC (Beckman-Coulter, Villepinte, France), Live-Dead blue (Thermo-Fisher, Scientific/Life-Technologies), Sorting was performed as described [47] on a 5 laser beams FACSAria- (Becton-Dickinson) in a BSL3 (CyPS platform) in order to separate: CD3-CD4+CD14+ Monocytes, activated CD25+CD69+HLADR+ and resting CD25-CD69-HLADR- CD3+4+ T cells. Resting CD4+ T cells were sub-sorted in CD45RA+CCR7+CD27+ Naïve (TN), CD45RA-CCR7+CD27+ Central-memory (TCM), CD45RA-CCR7-CD27+ Transitional-memory (TTM) and CD45RA-CCR7-CD27- Effector-memory (TEM) T cells, with purity above 95%. The median number of cells collected per subject was: 1.36, 0.27, 1, 1.36, 0.61, 0.55 and 0.12 million cells for monocytes, activated CD4 T-cells, resting CD4 T-cells, TN; TCM; TTM and TEM respectively. From each sorted subset an aliquot of 5,000 to 50,000 cells was cryopreserved for transcriptome analysis and immediately frozen in dry pellet at -80°C. All remaining sorting cells were used for HIV-2 DNA quantification.

### Quantification of HIV-2 total DNA

PBMC and dried pellets of each sorted fraction were stored at -80°C for HIV-2 DNA quantification. For PBMCs, total DNA was extracted from 3 to 5 million cells using the QIAsymphony DSP DNA mini kit (Qiagen, Courtaboeuf, France). For the CD4+ cell subsets, total DNA was extracted using QIAamp DNA Mini kit (Qiagen) when cells counted more than 500 000 cells (n = 57) and QIAamp DNA Micro kit (Qiagen) when cells counted less than 500 000 cells (n = 41). To normalize HIV-2 DNA quantification, the amount of total DNA in extracts was determined by quantification of the albumin gene using the LightCycler FastStart DNA Master Hybprobe kit (Roche) and serial dilutions of Human Genomic DNA (Roche) as standard [63, 64]. HIV-2 DNA was quantified using a real-time PCR assay with a 95% limit of detection (LOD) of 3 copies/PCR and a limit of quantification LOQ of 6 copies/PCR, showing good sensitivity and accuracy to quantify HIV-2 groups A and B [65]. The number of copies of HIV-2 DNA/μg total DNA was calculated using the extract concentration and the final results were reported as the number of copies/10^6^ cells. The formula used to convert these results was HIV-2 DNA copies/μg total DNA 1,000,000/150,000 HIV-2 DNA copies/10^6^ cells [66, 67].

When the HIV-2 DNA value was not quantifiable but a PCR signal was detectable, the results were arbitrarily assigned as an estimated value calculated as 50% of the HIV-2 DNA value, and expressed for one million cells, as done in other reservoir studies [48].

### HIV-2 *in vitro* reactivation assay

An average of 5 millions viable CD8-depleted PBMC from available samples of 11 HIV-2 infected subjects was cultured for 30 days in 10% FCS-supplemented RPMI 1640 medium after stimulation at day (D) 0 with anti-CD3/anti-CD28 + IL-2 (Sigma) + human recombinant IL-7 (R&D Systems) using a modified protocol from [47] with addition of IL-15 (Peprotech) simultaneously to other cytokines and of 5 millions allogeneic feeder CD8-depleted PBMC from a donor at D13. An average 1.5 million cells were collected at D3, D7, D10, and D14 for phenotypic analysis. Culture supernatants were collected every three days for HIV-2 viral load quantification at all time-points as described below. The viral production capacity was measured by using ultrasensitive pVL as described above and expressed as the ratio between the levels of HIV-2 RNA copies in supernatants at day of culture and HIV-2 DNA quantified in the corresponding CD8-depleted cells at D0 of culture [50].

### Flow cytometry analysis

A phenotypic analysis was performed during cell sorting. Additional phenotypic analyses of co-receptors and restriction factors were performed *ex vivo* on thawed PBMC from 7 HIV-2 infected individuals and donors either directly after thawing (D0) or after cell culture. The D0 cell surface staining was performed on thawed cells with: Fixable Viability-Stain-780, anti-CD3-BV510, -CD4-BUV395, -CD45RA-PE, -CCR7-PE-Cy7, -CD27-BUV737, -CD25-BV786, -HLA-DR-BV786, -CD69-BV786, -PD1-APC, -CXCR5-APC-R700, -CCR5-PE for expression on total CD4 cells or -CCR5-BV650 for expression on cell subsets (Becton-Dickinson). Cells were analyzed on a Fortessa flow cytometer (Becton-Dickinson) using the FacsDIVA version 6.1.3 software. The CD32, CXCR6 and TRIM5α expression was additionally studied at D0 after staining with: Viability FSV620, anti-CD3-APC-H7, -CD4-BV510, -CD45-RA-FITC, -CD27-PE-Cy7, -CCR7-APC-R700, -PD1-BB700, -CXCR5-AF647, -CXCR6-BV421, -CD32-PE (Becton-Dickinson). An indirect intra-cellular staining of TRIM5α was performed after permeabilization (CytoFix/CytoPerm, Becton-Dickinson). Briefly 1 million thawed cells were incubated on ice for 15 minutes with 5 μl anti-TRIM5α monoclonal antibody then washed twice and stained with the anti-IgG PE (both from Santa Cruz Biotechnology, CA, USA) then washed and incubated with the viability life-Dead then with CCR7 prior to membrane labeling. Cells were fixed (BD Cellfix) and run on the Gallios 3 Laser flow-cytometer and analyzed using FowJo (version 10.4.1). Phenotypic analysis was also performed on cultured cells on D3, D7, D10 and D13 using the D0 antibody panel except for -CCR5-BV650 and run on the Fortessa flow cytometer.

### Transcriptome analysis

Total RNA was extracted from each sorted TN, TCM, TTM, TEM and monocytes subset using a Mirvana kit (Life Technologies), treated with DNAse I (Life Technologies) and checked for purity on a ND-1000 spectrophotometer (NanoDrop-Technologies, Wilmington, Delaware, USA) before reverse transcription and gene-specific pre-amplification were performed (18 cycles). The cDNAs were then preamplified (PreAmp Master Mix, Fluidigm, Les Ulis, France) treated with exonuclease I (New-England Biolabs, Evry, France) and added to 96 forward and reverse primers matching genes involved in different pathways specific for T cells (S2 Table). The sequences of the primer pair oligos were validated in-house [68] or provided by DeltaGene (Fluidigm, South San Francisco, CA, USA) or Eurogentec (Eurogentec, Liège, Belgium) (S2 Table). Sample premix (Fast EvaGreen-Supermix; Bio- Rad, Marnes la Coquette, France) and assay premix were prepared for 96.96 Fluidigm Dynamic arrays (Fluidigm, San Fransisco, CA, USA). Two 96.96 Dynamic Array primed chips were then loaded and the preamplified prediluted cDNA samples. A real time-PCRs was run on a BioMark HD System for Genetic Analysis according to the Fluidigm Protocol. Invalid reactions, determined using the Real-time PCR-Analysis Software (Fluidigm), were treated as missing data. Data analysis of raw-Cq values were processed with Partek Genomics-Suite (Partek, Saint-Louis, Missouri, USA) as follows: *i)* principal-component analysis (PCA) was performed; *ii)* data normalization was completed by subtracting the value of the house-keeping gene RPS14 to the value of the target genes, *iii)* comparisons between TN, TCM, TTM and TEM subsets were done. The differentially expressed genes (DEGs) were identified by using the ANOVA program. Genes were considered differentially expressed if they met the following two criteria: a p-value of 0.05 and a ≥ 2 fold change.

### Statistical analysis

A two-tailed Wilcoxon matched-pairs signed rank test was used to compare cell subsets frequencies, CXCR6, TRIM5, IL22 and TP53 expression within TCM and TTM. The Mann-Whitney test and the Mc Nemar test were used to compare CD4 cells subsets in the different groups of individuals. Correlations between variables were determined with Spearman’s rank test. A p value lower than 0.05 was considered as significant. All values given in the text are medians and [IQR 25–75%]. Transcriptome was analyzed using Partek Genomics- Suite as above. Multivariate analysis allows analysis of all investigated genes. Multiple comparison correction was performed using a FDR of 0.05 [69].

## Supporting information

S1 Fig Expression of CCR5 on resting CD4+ T subsets: Naive (TN; CD45RA+CCR7+CD27+) central-memory (TCM; CD45RA-CCR7+CD27+) transitional memory (TTM; CD45RA-CCR7-CD27+) and effector-memory cells (TEM; CD45RA-CCR7-CD27-). Seven HIV-2 infected subjects (dot) were compared to 7 donors (D) (square). Each symbol represents a subject and the medians are shown.
(TIF)

S2 Fig Histograms of TRIM5α and IgG superimposed on total CD4 T-cells for HIV-2 infected subjects and donors (D)) and West-African donor (AD). (TIF)

S1 Table Description of number of cells assayed and of HIV-2 DNA copies in the different CD4 cell subsets. (DOCX)

S2 Table Primers used in transcriptomic studies. (DOCX)
