# Supplementary figures and images for: Limited HIV-2 reservoirs in central-memory CD4 T-cells associated to CXCR6 co-receptor expression in attenuated HIV-2 infection
Source: PLoS Pathog. 2019 May 16;15(5):e1007758. doi: 10.1371/journal.ppat.1007758 (PMC6541300; doi:10.1371/journal.ppat.1007758)

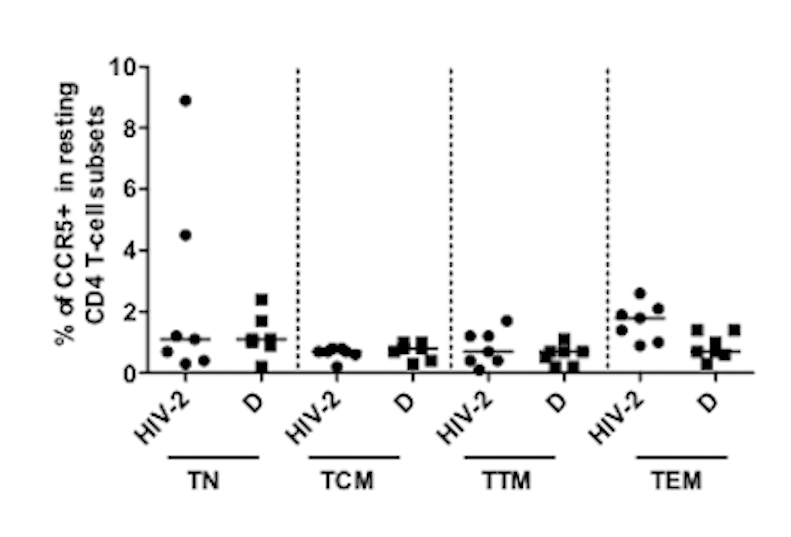

Supplement: S1 Fig — Seven HIV-2 infected subjects (dot) were compared to 7 donors (D) (square). Each symbol represents a subject and the medians are shown. (TIF) [file ppat.1007758.s001.tif]

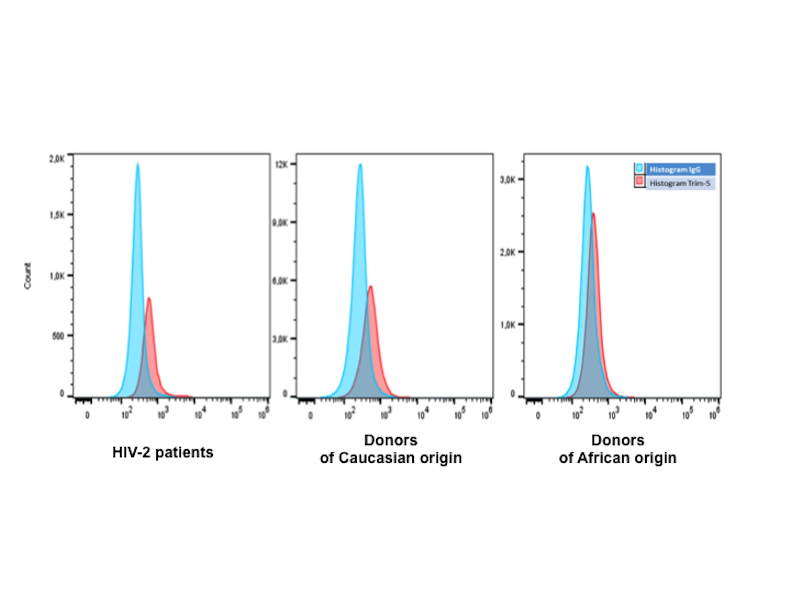

Supplement: S2 Fig — (TIF) [file ppat.1007758.s002.tif]
