# Supplementary material for: Limited HIV-2 reservoirs in central-memory CD4 T-cells associated to CXCR6 co-receptor expression in attenuated HIV-2 infection
Source: PLoS Pathog. 2019 May 16;15(5):e1007758. doi: 10.1371/journal.ppat.1007758 (PMC6541300; doi:10.1371/journal.ppat.1007758)
Supplement: S1 Table — (DOCX) [file ppat.1007758.s003.docx]

**S1 table: Description of number of cells assayed and of HIV-2 DNA copies in the different CD4 cell subsets.**

| **Participants** | **T naïve** | | **T central memory** | | **T transitional memory** | | **T effector memory** | | **Resting CD4 cells** | | **Activated CD4 cells** | | **Monocytes** | |
| --- | --- | --- | --- | --- | --- | --- | --- | --- | --- | --- | --- | --- | --- | --- |
|  | **Number**  **of cells (/PCR)** | **HIV-2 DNA (cp/PCR)** | **Number**  **of cells (/PCR)** | **HIV-2 DNA (cp/PCR)** | **Number**  **of cells (/PCR)** | **HIV-2 DNA (cp/PCR)** | **Number**  **of cells (/PCR)** | **HIV-2 DNA (cp/PCR)** | **Number**  **of cells (/PCR)** | **HIV-2 DNA (cp/PCR)** | **Number**  **of cells (/PCR)** | **HIV-2 DNA (cp/PCR)** | **Number of cells (/PCR)** | **HIV-2 DNA (cp/PCR)** |
| **1** | 41100 | No signal | 124800 | **7** | 81000 | 0.5 | 38700 | 0.5 | 102000 | **6** | 3000* | 0.5 | 178800 | No signal |
| **2** | 204000 | No signal | 61500 | 1 | 39000 | **7** | 24900* | 1 | 136800 | 1 | 68100 | 4 | 114900 | No signal |
| **3** | 18600* | No signal | 46500 | 3 | 56700 | **10** | 3000* | 0.5 | 72600 | 4 | 18000* | 2 | 37800 | No signal |
| **4** | 32100 | No signal | 56700 | 5 | 53700 | 2 | 21600* | No signal | 47100 | 0.5 | 30000 | No signal | 357000 | No signal |
| **5** | 25500* | No signal | 39000 | 2 | 33900 | 1 | 11400* | 0.5 | 37200 | 0.5 | 24000 | No signal | 81600 | No signal |
| **6** | 110100 | 0.5 | 81000 | 1 | 49500 | 1 | 23400* | No signal | 41400 | 0.5 | 54300 | No signal | 156300 | No signal |
| **7** | 74700 | No signal | 95100 | 0.5 | 86700 | 1 | 17700* | 0.5 | 150000 | 0.5 | 24000* | 0.5 | 150000 | No signal |
| **8** | 76200 | 0.5 | 57000 | 2 | 88200 | **7** | 18300* | 0.5 | 96900 | 1 | 45300 | 0.5 | 89700 | No signal |
| **9** | 26100* | No signal | 27300 | 0.5 | 63000 | 1 | 12900* | 0.5 | 70800 | 1 | 42000 | 1 | 24900* | No signal |
| **10** | 103500 | No signal | 56400 | 1 | 38400 | 1 | 6900* | No signal | 6000* | No signal | 87600 | 0.5 | 23400* | No signal |
| **11** | 69600 | No signal | 41100 | 0.5 | 56100 | 1 | 17700* | 0.5 | 101100 | 0.5 | 39300 | 0.5 | 84600 | No signal |
| **12** | 22800* | 0.5 | 57600 | 1 | 44100 | 4 | 29700 | 0.5 | 96000 | 4 | 18600* | 0.5 | 176700 | No signal |
| **13** | 111000 | No signal | 39300 | No signal | 34500 | No signal | 15000* | No signal | 91200 | No signal | 32400 | No signal | 133200 | No signal |
| **14** | 19800* | No signal | 71700 | 3 | 67200 | **100** | 5100* | 0.5 | 48600 | 2 | 19200 | 0.5 | 110400 | No signal |
|  |  |  |  |  |  |  |  |  |  |  |  |  |  |  |
| **Median** | 55350 |  | 56850 |  | 54900 |  | 17700 |  | 81900 |  | 31200 |  | 112650 |  |
| **IQR 25%** | 25650 |  | 42450 |  | 40275 |  | 11775 |  | 47475 |  | 20400 |  | 82350 |  |
| **IQR 75%** | 96675 |  | 60525 |  | 61425 |  | 20775 |  | 96675 |  | 44475 |  | 145800 |  |

***** DNA quantity < 200 ng/PCR ; cp: copies

The limit of quantification (LOQ) of the HIV-2 DNA assay is 6 copies/PCR; The limit of detection (LOD) of the HIV-2 DNA assay is 3 copies/PCR; A PCR signal can be detected below the LOD

Pt: patient; cp: copies
