# Supplementary material for: Limited HIV-2 reservoirs in central-memory CD4 T-cells associated to CXCR6 co-receptor expression in attenuated HIV-2 infection
Source: PLoS Pathog. 2019 May 16;15(5):e1007758. doi: 10.1371/journal.ppat.1007758 (PMC6541300; doi:10.1371/journal.ppat.1007758)
Supplement: S2 Table — (DOCX) [file ppat.1007758.s004.docx]

**S2 Table: Primers used in transcriptomic studies**

| **Target** | **Forward Primer** | **Reverse Primer** | **Design RefSeq** |
| --- | --- | --- | --- |
| APLNR | ACACGTACCGGGACTATGAC | CTGGCGTACATGTTGACGAA | NM_005161.N |
| APOBC3G | CGCAGCCTGTGTCAGAAAA | TTGCTCCAACAGTGCTGAAA | NM_021822.3 |
| BCL11B | CAACCCGCAGCACTTGTC | CCTCGTCTTCTTCGAGGATGG | NM_138576.2 |
| BCL6 | GATGGAGCATGTTGTGGACAC | AGGAGGCTTGATGGCAGAAA | NM_001706.4 |
| BRD4 | AGCTGGAGCGCTATGTCAC | GAGCCGGCAATCACATCAAC | NM_058243.2 |
| BST2 | AGAGAAGGCCCAAGGACAAA | AGACGCGTCCTGAAGCTTA | NM_004335.2 |
| CCL3 | ATGGCTCTCTGCAACCAGTT | CCGGGAGGTGTAGCTGAAG | NM_002983.2 |
| CCL4 | ACTATGAGACCAGCAGCCTCT | TCAGCACAGACTTGCTTGCT | NM_002984.3 |
| CCL5 | CCCTCGCTGTCATCCTCA | GGGCAATGTAGGCAAAGCA | NM_002985.2 |
| CCR1 | ACGGACAAAGTCCCTTGGAA | TCTGTGGTCGTGTCATAGTCC | NM_001295.N |
| CCR5 | TGAGACATCCGTTCCCCTACA | TGGCAGGGCTCCGATGTATA | NM_001100168.1 |
| CCR8 | GCAACACTGAAACCTCCAGAAC | ACTGTTGTCACACTGAGGTCAA | NM_005201.N |
| CD274 | GGAGATTAGATCCTGAGGAAAACCA | TGGAGGATGTGCCAGAGGTA | NM_014143.3 |
| CDKN1A /p21 | TGGAGACTCTCAGGGTCGAAAA | CGGCGTTTGGAGTGGTAGAA | NM_078467.1 |
| CX3CR1 | GTTGGTAGTGTTTGCCCTCAC | GGCCAGGTTCAGGAGGTAAA | NM_001171171.N |
| CXCL10 /IP10 | GCTGTACCTGCATCAGCATTA | CTGGATTCAGACATCTCTTCTCAC | NM_01565.2 |
| CXCR4 | ATCTTCCTGCCCACCATCTAC | CCCATGACCAGGATGACCAA | NM_003467.2 |
| CXCR5 | ATCTTCTTCCTCTGCTGGTCAC | GGTATTGTCCACGGCCTTCA | NM_001716.N |
| CXCR6 | TCTCTGGAACAAACTGGCAAA | CTGGCTGCTGTCATTGAAAC | NM_006564.N |
| DAP12 | ACCCGGAAACAGCGTATCA | TTGAGGTCGCTGTAGACATCC | NM_001173514.1 |
| DAXX | ATCTCGGAAGGAGAAGAAGCA | CACTGACCTTTGCCTTTCCA | NM_001141969.N |
| DDX3 | AGCTTCTTCAGTGATCGTGGAA | TGCCAATGCCATCGTAATCAC | NM_001193417.1 |
| DDX58 | GACTGGACGTGGCAAAACA | CTCCACTGGCTTTGAATGCA | NM_014314.3 |
| DNMT1 | GCCATTGGCTTGGAGATCA | AGCAGCTTCCTCCTCCTTTA | NM_001379.2 |
| DNMT3B | GTGAAGCACGAGGGGAATATCA | TTCCGCCAATCACCAAGTCA | NM_006892.3 |
| FOXO3 | CGCTCTCTCCGCTCGAA | TTTGCAGGGGCCACGTA | NM_001455.3 |
| GAG-Virus2 | GCGGAGAAACTCCGTCTTG | TTCGCTGCCCACACAATATGTT |  |
| GAPDH | ACACCATGGGGAAGGTGAAG | GTGACCAGGCGCCCAATA | NM_002046.3 |
| GPR1 | AAGGTACACCCAGGCATGAC | ACCTTGCTAAATGGAGAATGAAGAA | NM_001261453.N |
| GPR15 | GTGCAAAGGGAGCTCCTACA | TAGCGGTCAACACTCATGCA | NM_005290.N |
| HAVCR2 | GGATCCAAATCCCAGGCATAA | CTTGGAAAGGCTGCAGTGAA | NM_032782.3 |
| HIF1A | CAGTCGACACAGCCTGGATA | TTCTTCTGGCTCATATCCCATCAA | NM_001530.3 |
| IFI16 | GTGAATGGGGTGTTTGAGGTAC | CACCACTTCCATCTTCCCTGTA | NM_005531.2 |
| IFI27 | TTGTGGCTACTCTGCAGTCA | CCCAGGATGAACTTGGTCAA | NM_005532.3 |
| IFI44 | GGCTTTGGTGGGCACTAATA | TGCCATCTTTCCCGTCTCTA | NM_006417.4 |
| IFI6 | TGCTACCTGCTGCTCTTCA | TCAGGGCCTTCCAGAACC | NM_002038.3 |
| IFIT2 | AGAGTGCAGCTGCCTGAA | GTAGGCTGCTCTCCAAGGAA | NM_001547.4 |
| IFIT3 | ACTGGCAATTGCGATGTACC | GCTCAATGGCCTGCTTCAAA | NM_001549.4 |
| IFITM1 | ACACCCTCTTCTTGAACTGGT | CCAACCATCTTCCTGTCCCTA | NM_003641.3 |
| IFITM2 | ACTGAGAACCATCCCGGTAAC | CCGCTGTTGACAGGAGAGAA | NM_006435.2 |
| IFNA2 | TCTCCTGCTTGAAGGACAGAC | GGAACTGGTTGCCAAACTCC | NM_000605.3 |
| IFNAR1 | AGTGACGCTGTATGTGAGAAAA | ACGGGAGAGCAAATAATGCA | NM_000629.2 |
| IFNG | ACTGCCAGGACCCATATGTAA | GTTCCATTATCCGCTACATCTGAA | NM_000619.2 |
| IL17A | ACTACAACCGATCCACCTCAC | ACTTTGCCTCCCAGATCACA | NM_002190.2 |
| IL17F | CGCGTTTCCATGTCACGTA | CTGTACAACTTCCGAGGGGTA | NM_052872.3 |
| IL2 | ACCCAGGGACTTAATCAGCAA | GCATATTCACACATGAATGTTGTTTCA | NM_000586.3 |
| IL22 | TATCACCAACCGCACCTTCA | GTTTCTCCCCAATGAGACGAAC | NM_020525.4 |
| IL6 | AGAGCTGTGCAGATGAGTACAA | GTTGGGTCAGGGGTGGTTA | NM_000600.3 |
| IL7R | GGAGAAAGTGGCTATGCTCAA | CTGCGATCCATTCACTTCCA | NM_002185.2 |
| ILT7 | ACACAACGTCTCCTCCGAGT | TGTCCTGCGATCAGGATGT | NM_012276.3 |
| IRF1 | AACAAGGATGCCTGTTTGTTCC | TGGGATCTGGCTCCTTTTCC | NM_002198.2 |
| IRF2 | TGGCTGGAGGAGCAGATAAA | ATGCATCCAGGGGATCTGAA | NM_002199.3 |
| IRF3 | CCAGCCAGACACCTCTCC | TGGGGCCAACACCATGTTA | NM_001197122.1 |
| IRF4 | CTACAACCGCGAGGAGGAC | TGTCGATGCCTTCTCGGAAC | NM_002460.3 |
| IRF5 | AGATCTACGAGGTCTGCTCCAA | CCTCTCCTGCACCAAAAGAGTA | NM_002200.3 |
| IRF7 | GGCAGAGCCGTACCTGTCA | ACCGTGCGGCCCTTGTA | NM_004031.2 |
| IRF8 | TGGTCCAGGTCTTCGACAC | CGGCCCTGGCTGTTATAGAA | NM_002163.2 |
| IRF9 | TGCTCCAGGACTCCCTCAA | TGCTGCTCCCAATGTCTGAA | NM_006084.4 |
| ISG15 | CTGAGAGGCAGCGAACTCA | GCTCAGGGACACCTGGAA | NM_005101.3 |
| JUNB | TGGCCCAGCTCAAACAGAA | AGAAGGCGTGTCCCTTGAC | NM_002229.2 |
| LCK | ACCCACTGGTTACCTACGAA | TAGCTGTGCAGAGCGATAAC | NM_001042771.1 |
| LTR-17 | TAGTCGCCGCCTGGT | TTCCTGCCGCCCTTACT |  |
| MB21D1 | CTGAACACCGGGAGCTACTA | CTAGTTGAATTCTGGGGACTTCC | NM_138441.N |
| MDA-5 | GGTCTGGGGCATGGAGAATAA | CTGACACTTCCTTCTGCCAAAC | NM_022168.2 |
| MDM1 | CCATGGGCTGGACTTAGATCA | ATCTGTGGGTCATGGTAAGGG | NM_017440.4 |
| MDM2 | ACAGATTCCAGCTTCGGAAC | GCACCAACAGACTTTAATAACTTCA | NM_006881.3 |
| mTOR | CCAAACCCAGGTGTGATCAA | TCCTCATTTCCAGGCCACTA | NM_004958.3 |
| MX1 | ATGCTACTGTGGCCCAGAAA | GGCGCACCTTCTCCTCATA | NM_001144925.1 |
| MX2 | CGACTGGCAGAAAGACTTACC | CTTCTGGTGGCTCTCCCTTA | NM_002463.1 |
| NF-KB1 | CTGGAACCACGCCTCTAGATA | AAACTCTGGCTCATATGGTTTCC | NM_003998.3 |
| OAS3 New | GGATGGATGTTAGCCtggtg | CCTGACCCAGGACATTGAAG | NM_006187.2 |
| OASL | ACCATTGTGCCTGCCTACA | GGCCTTGATCAGGCTCACATA | NM_003733.3 |
| PDCD1/PD1 | CCCCAAGGCGCAGATCAA | CCAACCACCAGGGTTTGGAA | NM_005018.2 |
| PIK3R1 | CGAGTGGTTGGGCAATGAAAA | GTTGCTGCTTCCAACATTCCA | NM_181504.2 |
| PRDM1 | CCTGGTACACACGGGAGAAAA | TTGAGATTGCTGGTGCTGCTA | NM_001198.3 |
| PRKRA | TGACCCTTCCAAGCAACCAA | TCTCCAGCCATGATGAATAGCC | NM_001316362.N |
| RDC1 | GTCCCAACATGCCCAACAAA | GCAATCATGCCGATGACGAA | FLDM-078867.1 |
| RORg | CAAGACTCATCGCCAAAGCA | TTTCCACATGCTGGCTACAC | NM_001001523.1 |
| RPS14 | GCCCGCTCGGGTATGAA | TTCCTGCGAGTGCTGTCA | NM_001025071.1 |
| SAMHD1 | CCCGTGTCTGTGAAGTAGACAA | TGCGAGTGTGGAACATGTCA | NM_015474.3 |
| Scr | AGGAGCCCATTTACATCGTCAC | GCAGGTACTTGCCTGTCTCC | NM_005417.3 |
| SERINC3 | TCCCTCATCACCCTCTACACTA | TCAGGTTGGGATTGCAGGAA | NM_006811.N |
| SERINC5 | ATACTGGGGACCAGCCTCTTA | CCCTGCAGAGCGTCAGAA | NM_001174072.N |
| SOCS1 | CATCCGCGTGCACTTTCA | GCTCGAAGAGGCAGTCGAA | NM_003745.1 |
| STAT1 | TACCTGGCACAGTGGTTAGAA | TGAAAACGGATGGTGGCAAA | NM_007315.3 |
| STAT3 | GGAAATAATGGTGAAGGTGCTGAAC | CCGAGGTCAACTCCATGTCAAA | NM_003150.3 |
| STAT5 | CCCAGGCTCCCTATAACATGTA | ATGGTCTCATCCAGGTCGAA | NM_003152.3 |
| TBX21 | GGGCGTCCAACAATGTGAC | CCGTCGTTCACCTCAACGATA | NM_013351.1 |
| TIGIT | GTGGTGGTCGCGTTGACTA | TCCTGTCCAGCTGATTTTCTCC | NM_173799.3 |
| TLR7 | TCTTCAACCAGACCTCTACATTCC | AGCCCCAAGGAGTTTGGAAA | NM_016562.3 |
| TLR9 New | CCCCAGCATGGGTTTCTG | GAGAGACAGCGGGTGCAG | NM_017442.2 |
| TNF | TGTAGCAAACCCTCAAGCTG | ATTGGCCAGGAGGGCATT | NM_000594.2 |
| TP53 | TGAATGAGGCCTTGGAACTCA | TCAGGCCCTTCTGTCTTGAA | NM_000546.4 |
| TREX1 | ACCATCTGCTGTCACAACCA | TCTCTCCAAGGCTGGGACTA | NM_033629.3 |
| TRIM22 | CTCCCCTGATTCAAGACTCC | GCAGGTCACCTCCTTCTCTA | NM_006074.4 |
| TRIM5 | AGAAGCAGCAGGAAGCTGAA | GCCAAGACGTTGGTTTTGTCA | NM_033092.2 |
